# Impact of fatigue on activities of daily living in patients with rheumatoid arthritis: A cross-sectional study from the Aseer region, Saudi Arabia

**DOI:** 10.1097/MD.0000000000050372

**Published:** 2026-08-21

**Authors:** Khaled Abdulwahab Amer, Mansour Somaily, Abdulrahman Ali Aldosari, Lujane Mohammed Al-Tarish, Sereen Dhafer Al-Muhsin, Muhammed Alhussain Alkhairi, Fahad Saeed Thamer Alahmari, Abdussalam Mohammed A. Alqhtani

**Affiliations:** a Department of Internal Medicine, Aseer Central Hospital, Abha, Aseer, Saudi Arabia; b Department of Rheumatology, Armed Forces Hospital Southern Region, Khamis Mushait, Aseer, Saudi Arabia; c Department of Radiology, Aseer Central Hospital, Abha, Aseer, Saudi Arabia; d Khamis Mushait Maternity and Children’s Hospital, Aseer Health Cluster, Khamis Mushait, Aseer, Saudi Arabia.

**Keywords:** activities of daily living, BRAF-MDQ, DAS28-CRP, disease activity, fatigue, rheumatoid arthritis, Saudi Arabia

## Abstract

Fatigue is a prevalent and disabling feature of rheumatoid arthritis (RA) that interferes with everyday tasks and overall quality of life. Evidence on how RA-related fatigue affects activities of daily living (ADL) in Middle Eastern, and particularly Saudi Arabian, populations is sparse. This study aimed to quantify fatigue severity in patients with RA seen at a tertiary center in the Aseer region and examine its relationship with disease activity and ADL, using the Bristol RA Fatigue Multidimensional Questionnaire (BRAF-MDQ). We carried out a single-center, cross-sectional study with prospective data collection at the rheumatology outpatient clinics of Aseer Central Hospital, Saudi Arabia, between March and December 2024. Adults aged 16 years or older with RA, classified according to the 2010 American College of Rheumatology–European Alliance of Associations for Rheumatology criteria, were enrolled consecutively by convenience sampling. Fatigue was rated with the Arabic version of the BRAF-MDQ; disease activity was scored with the Disease Activity Score in 28 joints using C-reactive protein (DAS28-CRP); and functional status was captured with a validated 12-item ADL score. Independent-samples *t*-tests, Spearman rank correlation, and multivariable linear regression were performed in International Business Machines Statistical Package for the Social Sciences Statistics version 22 (IBM Corp.). A total of 140 patients were analyzed (82.1% women; mean age 47 years). Women had higher global BRAF-MDQ scores than men (46.1 vs 32.2; *P* < .001), with the difference driven mainly by the physical and emotional subscales. Patients in the high-fatigue group showed substantially greater ADL impairment than those in the low-fatigue group (10.64 ± 7.45 vs 3.42 ± 2.54; *P* < .001). BRAF-MDQ scores correlated strongly with both DAS28-CRP (*ρ* = 0.932–0.989) and ADL (*ρ* = 0.879–0.992). In the multivariable model, DAS28-CRP (*β* = 0.055; 95% confidence interval, 0.040–0.070) and ADL (*β* = 0.042; 95% confidence interval, 0.030–0.054) were each independently and positively associated with global fatigue. In this Saudi cohort, fatigue in RA was widespread and tracked closely with both disease activity and impairment of daily activities, with women reporting heavier burden in unadjusted comparisons. Routine fatigue assessment, together with evidence-based non-pharmacological strategies, should be embedded into the ambulatory care of patients with RA.

## 1. Introduction

Rheumatoid arthritis (RA) is a chronic, symmetrical inflammatory polyarthritis with a global prevalence of roughly 0.3 to 1.0% and a striking female predominance.^[1,2]^ Although joint involvement defines the disease, patients also contend with a broad spectrum of systemic symptoms, of which fatigue is among the most prevalent and the most distressing. RA-related fatigue is best understood as a persistent, overwhelming sense of physical and mental depletion that is disproportionate to exertion, is poorly relieved by rest, and arises from an interplay of inflammation, pain, sleep disturbance, depressed mood, functional disability, and psychosocial stressors.^[3–5]^

Fatigue is reported by 40 to 80% of patients with RA and exacts a notable toll on physical capacity, emotional well-being, social participation, and overall quality of life.^[6–8]^ Despite this, the symptom remains under-recognized in routine practice. Many patients describe coping with fatigue privately, viewing it as an inseparable part of their illness, while clinicians have historically treated it as a secondary concern, addressed mainly through generic advice on activity and rest.^[9,10]^

The 2023 European Alliance of Associations for Rheumatology (EULAR) recommendations for the management of fatigue in inflammatory rheumatic and musculoskeletal diseases have helped to reposition fatigue as a clinical priority, endorsing routine assessment and identifying tailored physical activity, cognitive behavioral therapy, and structured self-management education as first-line interventions, while acknowledging that pharmacological options targeting fatigue specifically remain limited.^[11]^ Earlier work has likewise framed fatigue in RA as a multidimensional construct with physical, behavioral, cognitive, and emotional facets, shaped by both disease-related drivers (inflammation, pain, disability) and contextual factors (mood, sleep, perceived social support).^[12–18]^

Although RA-related fatigue has been characterized extensively in international cohorts, dedicated evidence from Middle Eastern (and specifically Saudi Arabian) populations, although expanding, remains comparatively limited. Recent Saudi studies have begun to close this gap: Bawazir and Mustafa reported a high-fatigue burden closely tied to disease activity in a single-center cohort of patients with RA,^[19]^ and Alhashim and colleagues identified central white-matter (myelin) correlates of RA-related fatigue.^[20]^ Even so, few regional studies have quantified fatigue with a multidimensional instrument such as the Bristol RA Fatigue Multidimensional Questionnaire (BRAF-MDQ) or related it directly to activities of daily living (ADL). To address this gap, we set out to characterize fatigue severity in patients with RA attending a tertiary center in the Aseer region and to explore its association with disease activity (Disease Activity Score in 28 joints using C-reactive protein [DAS28-CRP]) and ADL, using the BRAF-MDQ

## 2. Materials and methods

### 2.1. Study design, setting, and sample size

**Figure 1.**
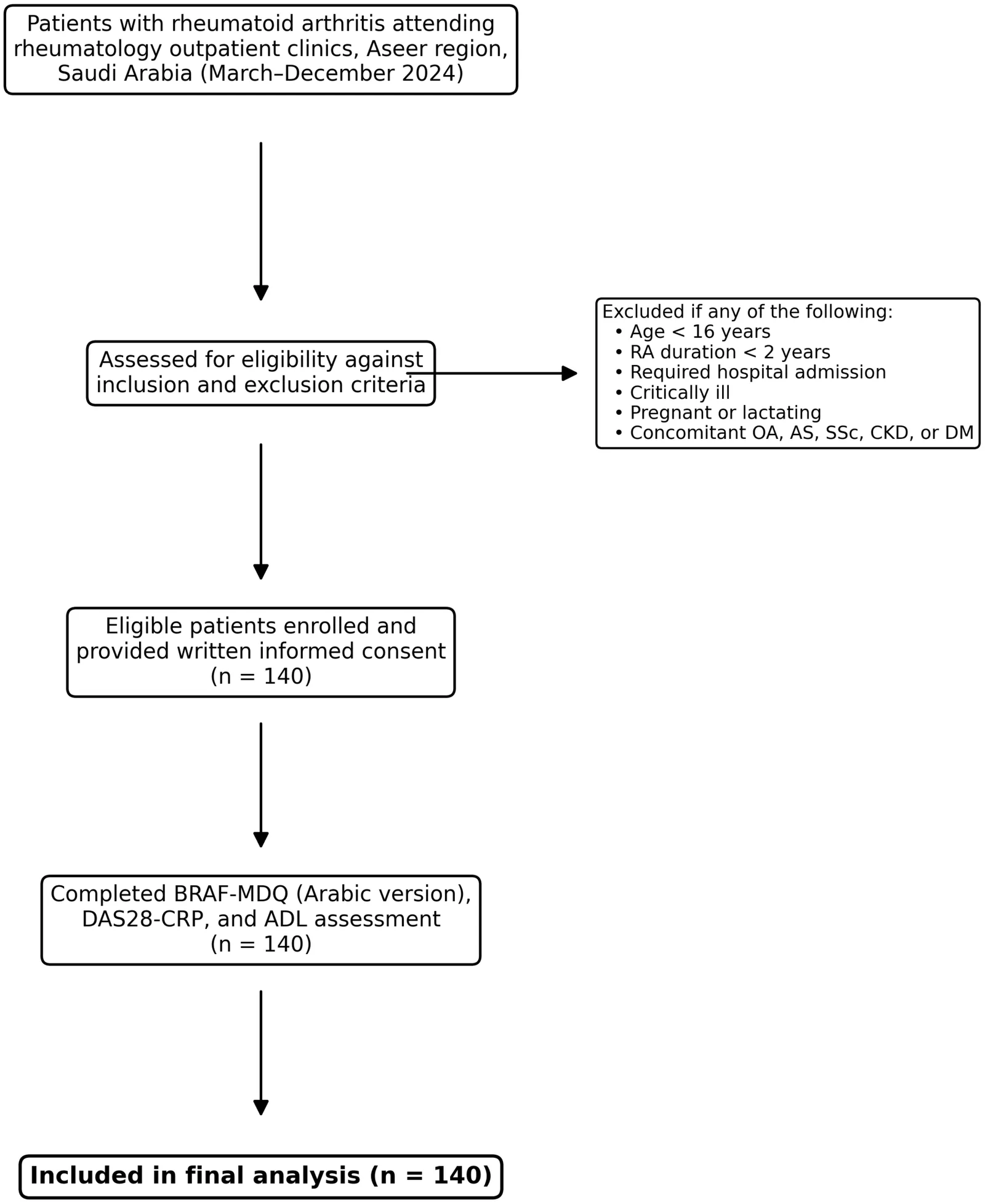
STROBE-compliant participant flow diagram, showing screening, eligibility, enrollment, assessment, and inclusion in the final analysis (N = 140). ADL = activities of daily living, AS = ankylosing spondylitis. BRAF-MDQ = Bristol Rheumatoid Arthritis Fatigue Multidimensional Questionnaire, CKD = chronic kidney disease, DAS28-CRP = Disease Activity Score in 28 joints using C-reactive protein, DM = diabetes mellitus, N/n = number of patients, OA = osteoarthritis, RA = rheumatoid arthritis, SSc = systemic sclerosis, STROBE = Strengthening the Reporting of Observational Studies in Epidemiology.

This was a single-center, cross-sectional study with prospective data collection, undertaken between March and December 2024 at the rheumatology outpatient clinics of Aseer Central Hospital and affiliated rheumatology services in the Aseer region, Saudi Arabia. During the 10-month recruitment window, every patient with RA presenting to the clinics who met the eligibility criteria was invited to take part, in keeping with a convenience sampling strategy. Participant flow is summarized in Figure 1.

A minimum sample of 97 patients was estimated using the standard single-proportion formula, assuming an expected fatigue prevalence of 50% in RA, a 95% confidence level, and 10% absolute precision. Recruitment continued until every eligible patient who attended the clinics within the prespecified window had been approached, yielding a final analytic sample of 140 patients, comfortably above the calculated minimum. Missing data were minimal because instruments were completed under research-team supervision; the only variable with incomplete information was disease duration, which was unavailable for 1 patient and is reported as such in Table 1.

**Table 1:** Baseline demographic and clinical characteristics of the study population (N = 140).

Characteristic	n	%
Age group (yrs)		
16–30	18	12.9
31–45	42	30.0
46–59	49	35.0
≥ 60	31	22.1
Sex		
Male	25	17.9
Female	115	82.1
Marital status		
Married	115	82.1
Unmarried	25	17.9
Educational level		
Primary school	64	45.7
Middle school	25	17.9
High school	17	12.1
Higher secondary	12	8.6
Graduate	22	15.7
Disease duration		
≤ 5 years	90	64.3
6–10 years	28	20.0
11–15 years	11	7.9
> 15 years	10	7.1
Missing	1	0.7
DAS28-CRP category		
Low (≤ 4.0)	36	25.7
Moderate (4.01–6.0)	69	49.3
High (> 6.0)	35	25.0

Mean age of the cohort = 47 years; mean DAS28-CRP = 4.88. Disease duration was unavailable for 1 patient (0.7%).

DAS28-CRP = Disease Activity Score in 28 joints using C-reactive protein, N/n = number of patients.

The study protocol received ethical approval from the Research Ethics Local Committee, College of Medicine, University of Bisha (registration number: H-06-BH-087), and was conducted in accordance with the Declaration of Helsinki. Written informed consent was obtained from every participant before any study procedure. Reporting follows the Strengthening the Reporting of Observational Studies in Epidemiology guideline for observational research.

### 2.2. Inclusion and exclusion criteria

Eligible participants were: aged 16 years or older; had an established diagnosis of RA of at least 2 years’ duration, fulfilling the 2010 American College of Rheumatology–EULAR classification criteria; attending the rheumatology outpatient clinics during the recruitment window; and able and willing to provide written informed consent.

Patients were excluded if they: required hospital admission or were critically unwell at the time of assessment; were pregnant or breastfeeding; or carried a concomitant diagnosis of osteoarthritis, ankylosing spondylitis, systemic sclerosis, chronic kidney disease, or diabetes mellitus, each of which could independently influence fatigue or function.

Other recognized contributors to fatigue (such as anemia, hypothyroidism, vitamin D deficiency, and malnutrition) were not used as prespecified exclusion criteria and were not screened for systematically at enrollment. The implications of this choice for residual confounding are addressed in the Limitations section.

### 2.3. Data collection and instruments

A structured case-report form was used to record demographic and clinical data, including age, sex, marital status, occupation, educational level, duration of RA, current medications, and comorbidities. Every participant underwent a focused clinical examination by the study physicians.

Disease activity was quantified with the DAS28-CRP, calculated through a validated online calculator from the 28-joint tender joint count, the 28-joint swollen joint count, and the serum C-reactive protein concentration, together with a patient global assessment, in line with the standard formula. For descriptive purposes, DAS28-CRP values were categorized as low (≤ 4.0), moderate (4.01–6.0), or high (> 6.0).

Fatigue was assessed with the Arabic version of the BRAF-MDQ, a 20-item patient-reported instrument that yields a global score (range 0–70) alongside 4 subscale scores: physical fatigue (0–22), living with fatigue (0–21), cognitive fatigue (0–15), and emotional fatigue (0–12). Higher scores denote greater fatigue burden.^[21]^

To ensure conceptual equivalence between the original and Arabic versions of the BRAF-MDQ, an independent certified translator completed a forward translation, which was then back-translated into English by a second, blinded translator; discrepancies were reconciled by consensus. Face validity was reviewed by a medical sociology faculty member familiar with patient-reported outcome measures. Before formal data collection, the translated questionnaire was pilot-tested with a small group of Arabic-speaking patients with RA to confirm clarity, cultural acceptability, and practical reliability of the wording.

Functional status was captured with the Lawton–Brody ADL scale, a validated 12-item instrument covering basic self-care and instrumental tasks that has been widely used in chronic disease populations; higher scores reflect greater functional impairment.^[22]^

### 2.4. Statistical analysis

All analyses were performed in International Business Machines Statistical Package for the Social Sciences Statistics version 22 (IBM Corp.). Continuous variables are summarized as mean ± standard deviation, and categorical variables as counts and percentages. Two-sided tests were used throughout, with *P* < .05 considered statistically significant. Independent-samples *t*-tests compared BRAF-MDQ scores between male and female patients. For descriptive comparisons of ADL impairment, patients were classified as having high fatigue if their global BRAF-MDQ score was 35 or above (the midpoint of the 0–70 scale) and low fatigue if their score was below 35. Spearman rank correlation coefficients were used to examine the strength and direction of associations between BRAF-MDQ dimensions, DAS28-CRP, and ADL scores. A multivariable linear regression model was fitted with global BRAF-MDQ score as the dependent variable. DAS28-CRP and ADL score were selected as independent variables on the basis of their established associations with RA fatigue in the prior literature. The remaining collected variables (age, sex, disease duration, educational level, and medications) were not included in the primary model, as none reached the prespecified entry threshold of *P* < .20 in univariate screening, and their inclusion did not materially alter the point estimates for DAS28-CRP or ADL.

## 3. Results

**Figure 2.**
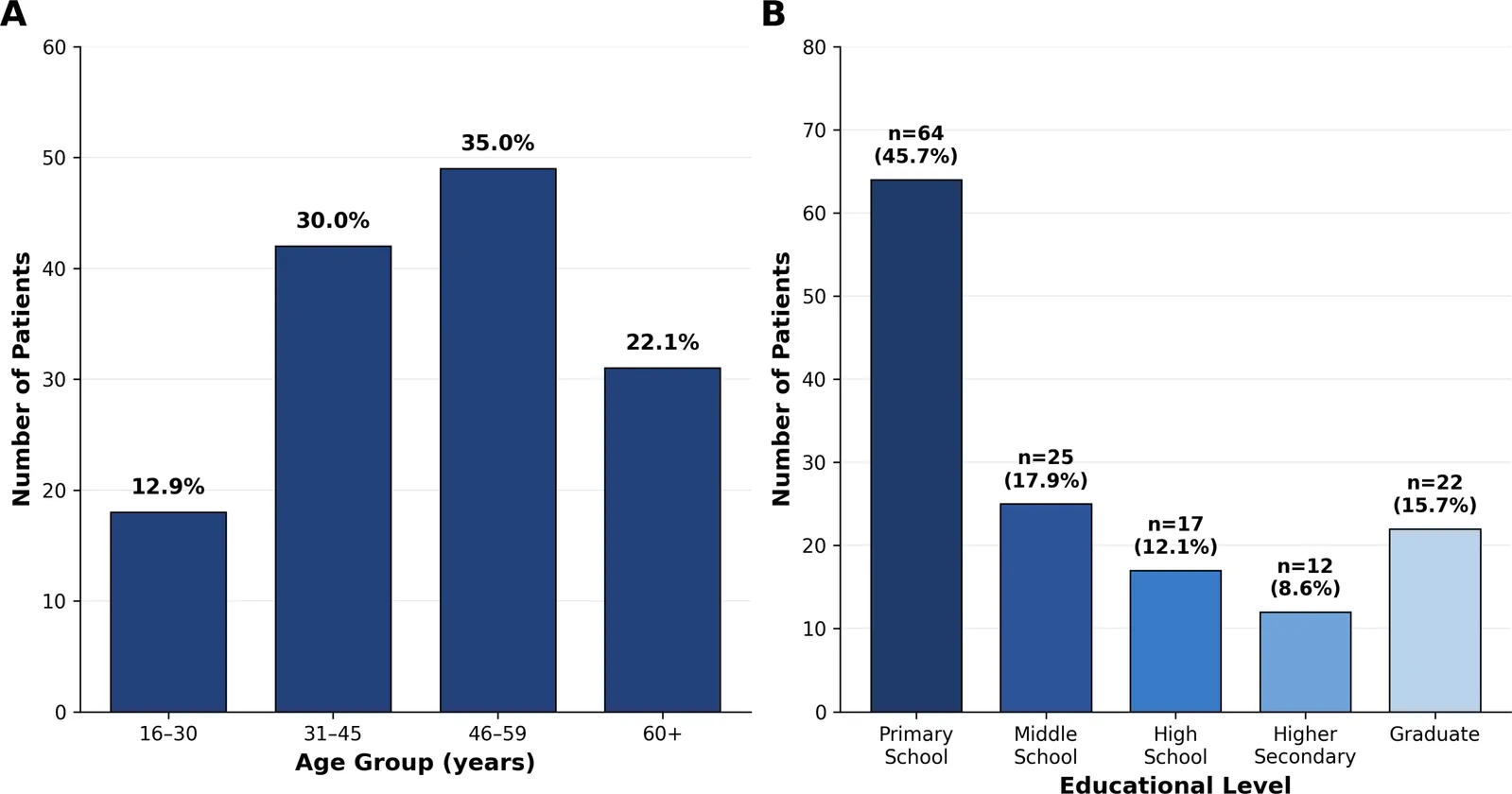
Distribution of (A) age and (B) educational level among patients with RA included in the study (N = 140). N/n = number of patients, RA = rheumatoid arthritis

Of all eligible patients who attended the clinics during the recruitment window, 140 were enrolled and contributed complete BRAF-MDQ, DAS28-CRP, and ADL data (Fig. 1). Baseline demographic and clinical features are summarized in Table 1. The cohort was predominantly female (115 of 140; 82.1%) and of middle age (mean 47 years), and most participants were married. Among the women, the majority described themselves as homemakers. Age and educational level distributions are shown in Figure 2.

Disease duration clustered in the early years: 90 patients (64.3%) had lived with RA for 5 years or fewer, 28 (20.0%) for 6 to 10 years, 11 (7.9%) for 11 to 15 years, and 10 (7.1%) for more than 15 years (duration unavailable for 1 patient). The mean DAS28-CRP was 4.88; on this basis, 36 patients (25.7%) were classified as having low-disease activity, 69 (49.3%) as moderate, and 35 (25.0%) as high (Table 1).

**Figure 3.**
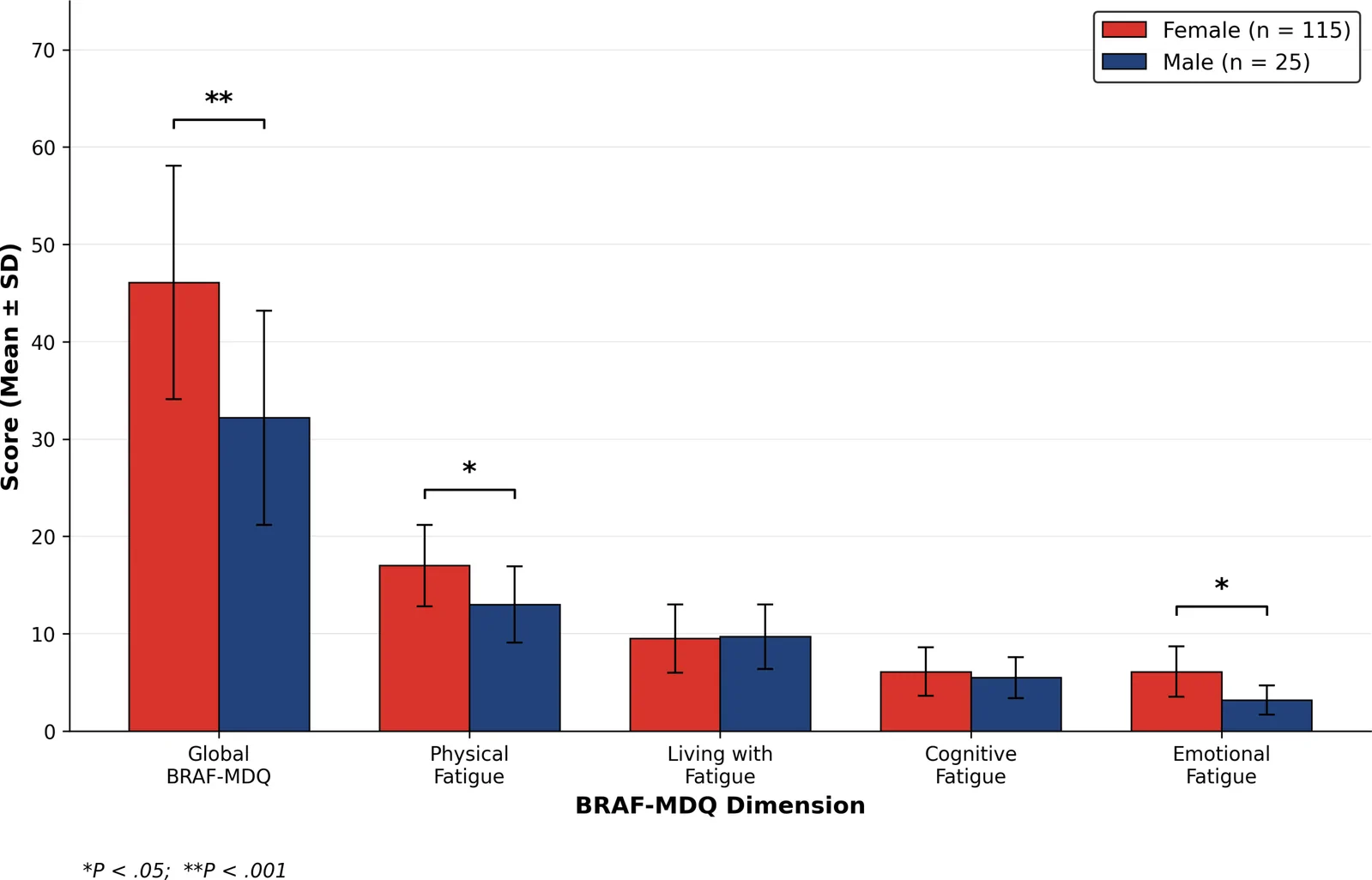
BRAF-MDQ fatigue scores by sex across the global score and the 4 subscales in patients with RA. *P < .05; **P < .001. BRAF-MDQ = Bristol Rheumatoid Arthritis Fatigue Multidimensional Questionnaire, n = number of patients, RA = rheumatoid arthritis, SD = standard deviation.

Comparisons of BRAF-MDQ scores by sex are displayed in Figure 3. Female patients reported significantly higher global BRAF-MDQ scores than men (46.1 vs 32.2; *P* < .001), with statistically significant differences confined to the physical (17.0 vs 13.0; *P* < .05) and emotional (6.1 vs 3.2; *P* = .024) subscales. The cognitive (6.1 vs 5.5; *P* = .50) and living-with-fatigue (9.5 vs 9.7; *P* = .90) subscales did not differ between sexes.

Patients in the high global BRAF-MDQ group exhibited markedly greater ADL impairment than those in the low group (10.64 ± 7.45 vs 3.42 ± 2.54; *P* < .001; Table 2). Spearman correlation coefficients linking each BRAF-MDQ dimension with DAS28-CRP and the ADL score are detailed in Table 3. The total BRAF-MDQ score correlated very strongly with DAS28-CRP (*ρ* = 0.989; *P* < .001) and with ADL (*ρ* = 0.988; *P* < .001), and every subscale-level correlation was statistically significant (all *P* < .001).

**Table 2:** ADL score in patients with high and low global BRAF-MDQ fatigue scores.

Group	ADL Score (Mean ± SD)	*P* value
High BRAF-MDQ score	10.64 ± 7.45	< .001
Low BRAF-MDQ score	3.42 ± 2.54	

High fatigue: global BRAF-MDQ score ≥ 35 (midpoint of the 0–70 scale); low fatigue: global BRAF-MDQ score < 35.

ADL = activities of daily living, BRAF-MDQ = Bristol Rheumatoid Arthritis Fatigue Multidimensional Questionnaire, SD = standard deviation.

**Table 3:** Spearman correlation coefficients (*ρ*) between BRAF-MDQ fatigue dimensions, DAS28-CRP, and ADL score.

BRAF-MDQ dimension	*ρ* (DAS28-CRP)	*P* value (DAS28-CRP)	*ρ* (ADL)	*P* value (ADL)
Total	0.989	< .001	0.988	< .001
Physical	0.982	< .001	0.889	< .001
Living with fatigue	0.968	< .001	0.879	< .001
Cognitive	0.967	< .001	0.992	< .001
Emotional	0.932	< .001	0.965	< .001

All values were verified against the original Statistical Package for the Social Sciences output. The high magnitude of the coefficients is discussed in the Discussion section.

ADL = activities of daily living, BRAF-MDQ = Bristol Rheumatoid Arthritis Fatigue Multidimensional Questionnaire, DAS28-CRP = Disease Activity Score in 28 joints using C-reactive protein.

In the multivariable linear regression model (Table 4), DAS28-CRP (*β* = 0.055; 95% confidence interval, 0.040–0.070; *P* < .001) and ADL score (*β* = 0.042; 95% confidence interval, 0.030–0.054; *P* < .001) were each independently and positively associated with global BRAF-MDQ fatigue, indicating that more active disease and greater difficulty with daily activities each contributed, after adjustment, to higher fatigue scores.

**Table 4:** Multivariable linear regression of global BRAF-MDQ score on DAS28-CRP and ADL score in patients with RA.

Variable	*β*	95% CI	*P* value
DAS28-CRP	0.055	0.040–0.070	< .001
ADL score	0.042	0.030–0.054	< .001

Dependent variable: global BRAF-MDQ score.

ADL = activities of daily living, BRAF-MDQ = Bristol Rheumatoid Arthritis Fatigue Multidimensional Questionnaire, CI = confidence interval, DAS28-CRP = Disease Activity Score in 28 joints using C-reactive protein, RA = rheumatoid arthritis.

## 4. Discussion

In this cross-sectional study with prospective data collection of 140 patients with RA from a tertiary center in the Aseer region, fatigue measured by the Arabic BRAF-MDQ was widespread and tracked closely with both disease activity (DAS28-CRP) and impairment of ADL. Both predictors retained their independent association with global fatigue in multivariable regression, underscoring that fatigue is a clinically meaningful axis of RA burden in this Saudi population, not an incidental symptom.

RA-related fatigue is a long-recognized, persistent, and multicausal symptom that erodes patients’ ability to handle self-care, employment, and social roles, with measurable consequences for physical, mental, and emotional health.^[8,23–25]^ It accounts for a large share of the perceived decline in general health reported by people living with RA.^[25]^ Yet, unlike pain or joint damage, fatigue has rarely been treated as a distinct therapeutic target in routine care.^[26]^ Our results align with prior cohorts that linked fatigue severity to disease activity and functional impairment^[27,28]^ and extend those observations to a Saudi RA population, for whom such data have until now been thin on the ground.

Female patients reported higher global, physical, and emotional BRAF-MDQ scores than men. These are unadjusted comparisons drawn from a markedly imbalanced sample (82% female, 18% male) and were not retested in a sex-stratified multivariable model; the difference should therefore be read as hypothesis-generating rather than confirmatory. The literature on sex and RA fatigue is mixed: some cohorts, including the work of Belza et al,^[29]^ have likewise observed a heavier fatigue burden in women, while others have reported that apparent sex differences attenuate after adjustment for disease activity, pain, and psychosocial covariates. The small male subgroup in our sample (n = 25) limits the strength of any conclusion on this point.

The correlations we observed between BRAF-MDQ dimensions and both DAS28-CRP and ADL were significant and in the expected positive direction, consistent with previous reports linking RA fatigue severity to active inflammation, disability, and reduced productivity at work. The magnitude of the total BRAF-MDQ correlations (*ρ* = 0.989 with DAS28-CRP; *ρ* = 0.988 with ADL) is higher than those typically reported in the RA fatigue literature. Olsen et al (2016) observed correlations between fatigue and disease activity in the range of *ρ* ≈ 0.30 to 0.45 in a low-disease-activity cohort, and Badak SÖ (2022) reported BRAF-MDQ correlations with clinical measures of *ρ* = 0.40 to 0.65. The elevated values in our sample were verified against the original Statistical Package for the Social Sciences output and are reported as computed. They may partly reflect the compressed distributional range of a convenience sample in which nearly half the patients (49.3%) clustered in the moderate DAS28-CRP band (4.01–6.0), reducing within-sample variance and potentially inflating Spearman rank-order coefficients. Replication in larger, multicenter cohorts with greater disease-activity heterogeneity is warranted before the full magnitude of these associations can be definitively established.^[14,27,28]^ At the same time, earlier studies suggest that the capacity to live with fatigue improves with disease duration and the availability of psychosocial resources, implying that fatigue is shaped not only by biological disease characteristics but also by behavioral and social factors.^[13,30]^

Our findings also align with a small but expanding body of regional and mechanistic evidence. In a recent single-center Saudi cross-sectional study of 253 patients with RA at King Abdulaziz University Hospital, Bawazir and Mustafa found fatigue to be highly prevalent (reported by 80% of patients) and strongly and inversely related to disease activity, with a correlation of *r* = −0.68 between the Clinical Disease Activity Index and the Functional Assessment of Chronic Illness Therapy–Fatigue score in the high-activity subgroup, and with female sex and longer disease duration as independent correlates. This closely parallels both the disease-activity association and the heavier fatigue burden among women observed in our cohort.^[19]^ They also noted that fatigue persisted in patients whose inflammation was well controlled, reinforcing the view that RA-related fatigue is multifactorial rather than a simple function of disease activity. A neurobiological basis for this residual fatigue is suggested by Alhashim and colleagues, who used magnetization-transfer imaging to demonstrate reduced cerebral myelin integrity in patients with RA-related fatigue (most marked in the splenium of the corpus callosum and the middle cerebellar peduncle) changes that were independent of current disease-activity status.^[20]^ The functional and quality-of-life burden captured here through ADL impairment is likewise consistent with Dar et al, who reported substantially higher multidimensional fatigue scores in patients with RA than in controls (global fatigue index 33.2 vs 14.4) and significant inverse correlations between fatigue and the physical and psychological domains of quality of life.^[31]^ Together, these studies place our BRAF-MDQ and ADL findings within a coherent regional and international framework, while our data contribute a specifically multidimensional, ADL-anchored perspective from the Aseer region.

A meaningful response to fatigue therefore requires more than tighter control of inflammation. The 2023 EULAR recommendations stress that, while optimizing disease-modifying therapy is foundational, the strongest evidence for fatigue improvement supports non-pharmacological interventions (especially individually tailored aerobic and resistance exercise, cognitive behavioral therapy, and structured self-management education) whereas dedicated pharmacological anti-fatigue agents have produced inconsistent or modest effects beyond those attributable to disease control alone.^[11]^ Earlier randomized and observational studies in RA also point to exercise programs and self-efficacy–oriented self-management courses as effective levers for fatigue.^[30,32]^ Our findings reinforce the case for embedding routine fatigue screening (ideally with a validated tool such as the BRAF-MDQ) and these evidence-based interventions into ambulatory RA care, not least in Middle Eastern settings where fatigue has historically been overshadowed by joint-focused outcomes.

Several limitations deserve emphasis. First, the cross-sectional design precludes causal inference; the strong correlations between disease activity, ADL impairment, and fatigue should be interpreted with that caveat in mind. Second, recruitment was by convenience from a single tertiary center in 1 Saudi region, which constrains the wider generalizability of the findings. Third, common secondary contributors to fatigue (anemia, hypothyroidism, vitamin D deficiency, and malnutrition) were not used as exclusion criteria nor screened for systematically, so residual confounding from these conditions cannot be excluded. Fourth, sex-based comparisons are unadjusted and arise from a predominantly female sample. Finally, although the Arabic BRAF-MDQ used here was developed through forward–backward translation, face validation, and pilot testing, a formal published psychometric validation in a Saudi RA population is not yet available. Multicenter studies with larger and more balanced samples, comprehensive secondary-cause work-up, and formal psychometric validation of the Arabic instrument are warranted.

## 5. Conclusion

In this Saudi cohort, fatigue in RA was both common and tightly linked to disease activity and impairment of ADL. Routine screening for fatigue using validated instruments such as the BRAF-MDQ should be incorporated into the ambulatory care of patients with RA, and evidence-based non-pharmacological interventions (tailored exercise, cognitive behavioral therapy, and structured self-management education) should be offered alongside optimization of disease-modifying therapy.

## Acknowledgments

The authors thank the rheumatology clinic staff at Aseer Central Hospital for their support during data collection.

## Author contributions

**Conceptualization:** Khaled Abdulwahab Amer, Mansour Somaily.

**Data curation:** Abdulrahman Ali Aldosari, Lujane Mohammed Al-Tarish, Sereen Dhafer Al-Muhsin, Muhammed Alhussain Alkhairi, Fahad Saeed Thamer Alahmari, Abdussalam Mohammed A. Alqhtani.

**Methodology:** Abdulrahman Ali Aldosari.

**Investigation:** Lujane Mohammed Al-Tarish.

**Resources:** Muhammed Alhussain Alkhairi.

**Software:** Khaled Abdulwahab Amer, Sereen Dhafer Al-Muhsin.

**Supervision:** Mansour Somaily.

**Visualization:** Sereen Dhafer Al-Muhsin, Fahad Saeed Thamer Alahmari, Abdussalam Mohammed A. Alqhtani.

**Validation:** Abdussalam Mohammed A. Alqhtani.

**Writing – original draft:** Khaled Abdulwahab Amer.

**Writing – review & editing:** Khaled Abdulwahab Amer.
